# Adsorptive Removal of Arsenite and Cobalt by Commercial Sorbents

**DOI:** 10.3390/ma18225133

**Published:** 2025-11-12

**Authors:** Sevda Joudiazar, Sushma Yadav, Zhiming Zhang, Anshuman Satpathy, Eustace Fernando, Roxana Rahmati, Junchul Kim, Rupali Datta, Dibyendu Sarkar

**Affiliations:** 1 Department of Civil, Environmental and Ocean Engineering, Stevens Institute of Technology, Hoboken, NJ 07030, USA; sjoudiaz@stevens.edu (S.J.); fsushma@stevens.edu (S.Y.); satpathyanshuman92@gmail.com (A.S.); eustace6192@gmail.com (E.F.); rrahmati@stevens.edu (R.R.); 2 Department of Civil and Environmental Engineering, Rowan University, Glassboro, NJ 08028, USA; zhangz@rowan.edu; 3 Tetra Tech, Inc., King of Prussia, PA 19406, USA; jc.kim@tetratech.com; 4 Department of Biological Sciences, Michigan Technological University, Houghton, MI 49931, USA; rupdatta@mtu.edu

**Keywords:** arsenic, cobalt, adsorption, desorption, groundwater remediation

## Abstract

Despite the prevalence and toxicity of heavy metals in the environment, arsenic and cobalt are of particular concern due to their high mobility and bioaccumulation potential, particularly in contaminated groundwater. Herein, we studied the adsorption behavior of commercially available sorbents, including Fluorosorb-100 (FS-100), Fluorosorb-200 (FS-200), and Filtrasorb-400 (F-400), for the removal of arsenite (As(III)) and cobalt (Co(II)), aiming at the selection of filter media in terms of future groundwater remediation. Kinetic analysis revealed that As(III) adsorption followed a pseudo-second-order model, while Co(II) showed mixed first- and second-order behavior, reflecting sorbent-dependent mechanisms. Equilibrium isotherm modeling revealed strong correlations with both Langmuir and Freundlich models, confirming heterogeneous adsorption sites and multilayer interactions. FS-100 demonstrated the highest affinity for As(III) (qₘ = 0.46 mg/g) and F-400 exhibited the greatest adsorption capacity for Co(II) (qₘ = 1.00 mg/g), while FS-200 consistently showed relatively weaker adsorption for both metals. Desorption studies indicated predominantly irreversible binding, with minimal release of As(III) from F-400 and Co(II) from FS-200 and F-400, even at high concentrations. Overall, these findings highlight that commercially available sorbents can effectively capture arsenite and cobalt, offering cost-effective and scalable options for heavy-metal removal in groundwater remediation systems under realistic environmental conditions.

## 1. Introduction

Groundwater contamination is a pervasive and growing environmental challenge, largely driven by industrial, agricultural, and urban activities that introduce toxic pollutants into aquatic systems, posing significant risks to ecosystems and human health [1,2]. Among these pollutants, heavy metals are among the most concerning contaminants in domestic, industrial, and agricultural wastewaters due to their toxicity, persistence, and potential for bioaccumulation [3,4]. Arsenic (As) and cobalt (Co) are especially significant owing to their high mobility and severe health implications [5,6]. Arsenic, a recognized human carcinogen, is widely reported in groundwater worldwide, where it predominantly exists in inorganic forms arsenate (As(V)) and arsenite (As(III)), with As(III) being more toxic and mobile [7,8]. Consequently, the World Health Organization (WHO) has established a maximum guideline value of 0.01 mg/L for arsenic in drinking water [9]. Cobalt, though an essential trace element, becomes toxic at elevated concentrations and can cause thyroid dysfunction and respiratory disorders. Its persistence and accumulation in groundwater further exacerbate ecological and health concerns [10,11,12]. Given the toxicity, persistence, and environmental mobility of these metals, developing efficient and sustainable remediation strategies for groundwater is of critical importance.

A wide range of conventional treatment technologies has been explored for the remediation of metal-contaminated waters, including chemical precipitation [13], ion exchange [14], membrane separation [15], and adsorption [3,16]. Among these, adsorption is particularly attractive for groundwater remediation due to its cost-effectiveness, operational simplicity, and applicability in permeable reactive barriers (PRBs) for in situ contaminant capture [8,17,18,19]. The performance of PRBs largely depends on the physicochemical characteristics of the reactive media, including surface area, pore structure, and surface chemistry, which determine both removal efficiency and long-term stability [20,21,22]. Field-oriented research has also shown that system configuration and local geochemical conditions, including pH and competing ions, strongly affect the overall performance of a reactive remediation system [23]. Therefore, selecting an appropriate sorbent tailored to the specific contaminant is a key factor in achieving effective adsorption performance in PRBs system [18].

With advances in novel sorbents, researchers have been extensively studying the adsorption of As(III) and Co(II) using various sorbents with a few examples listed in Table 1. Asere et al. have compared the use of natural sorbents for arsenic remediation and their limitations, such as low metal ion uptake, which usually requires a surface coating or functionality to improve their affinity for metal uptake [24]. Considering the drawbacks of natural sorbents, the cost-effectiveness, established performance, widespread availability, and large-scale in situ remediation efforts make commercially available sorbents a preferred choice for treating contaminated groundwater [25]. One of the most widely used commercial sorbents, granular activated carbon (GAC), has proven highly effective for removing both organic and inorganic contaminants from water [24,25,26], as it captures heavy metals primarily through electrostatic interactions and a complexation mechanism [27]. Although activated carbon is a highly effective sorbent, its application is limited by cost and exhaustion after use; therefore, recent efforts have focused on developing modified forms with improved adsorption performance [28]. In addition to conventional activated carbon, other carbon-based materials such as biochars, graphene oxide, and reduced graphene oxide have gained increasing attention for metal ion removal. Biochars are lower-cost and more sustainable carbonaceous alternatives that can be effective for metal removal, particularly when surface chemistry is tailored by activation or metal/iron functionalization [28], but their native adsorption capacity is often limited, and improving it requires surface functionalization, optimization of production methods and dosages, and careful consideration of regeneration and economic feasibility for large-scale applications [29]. Graphene-based adsorbents, on the other hand, exhibit exceptionally high surface area and abundant oxygen-containing functional groups, enabling strong binding with metal ions [30,31]. However, despite their high adsorption capacity and reusability, graphene oxide composites are generally more expensive than conventional adsorbents like activated carbon or zeolite [32]. In addition to carbonaceous materials, clays and clay minerals are abundant adsorbents that have been utilized for decades, both in their natural and modified forms, to effectively remove a wide range of toxic heavy metals from aqueous solutions [33]. Clay exhibits high surface area, tunable hydrophobicity, and strong ion-exchange capacity, making them promising candidates for PRB systems [34]. To further enhance their adsorption of organic and inorganic contaminants, clays can be chemically modified into organoclays. These materials, prepared by intercalating clays with cationic, nonionic, or zwitterionic surfactants, have been shown to adsorb cationic and anionic organic compounds, hydrophobic molecules, and inorganic anions through ion exchange, ion–dipole, and hydrophobic interactions, making them versatile sorbents for water remediation in both batch and percolation systems [35,36].

In this study, we evaluated the performance of commercially available organoclays (Fluorosorb-100 (FS-100) and Fluorosorb-200 (FS-200)), and granular activated carbon (Filtrasorb-400 (F-400)), for the removal of arsenite and cobalt. Results from adsorption isotherms, kinetic, and desorption experiments provided critical insights into the effectiveness and potential limitations of these sorbents for arsenite and cobalt remediation. Furthermore, the findings provided practical guidance for selecting suitable reactive media in future applications, particularly for deployment in permeable reactive barrier (PRB) systems for groundwater remediation.

## 2. Materials and Methods

### 2.1. Materials

Sodium nitrate (NaNO_3_, Fisher Scientific, Waltham, MA, USA) was employed as the background electrolyte in all adsorption and desorption experiments. Solution pH was adjusted using diluted nitric acid (HNO_3_, 68–70%, Fisher Scientific, Waltham, MA, USA) and sodium hydroxide (NaOH, 98%, Fisher Scientific, Waltham, MA, USA). Arsenic standards were prepared by diluting a 1000 μg/mL arsenic oxide in 4% HNO_3_ (Plasma Cal, New York, NY, USA), while cobalt standards were obtained from a 1 mg/mL cobalt nitrate solution in 2% HNO_3_. Synthetic arsenite solutions were prepared from sodium arsenite (NaAsO_2_), and cobalt solutions were derived from cobalt nitrate hexahydrate (Co(NO_3_)_2_·6H_2_O). All reagents were of analytical grade, and solutions were prepared using ultrapure (Millipore, Kalamazoo, MI, USA) water.

The commercial sorbent materials (FS-100, FS-200, and F-400) were supplied by Tetra Tech Inc. (King of Prussia, PA, USA). All sorbents were used as received from the manufacturers without additional pre-conditioning, washing, or modification. The physicochemical properties of these sorbents were previously characterized in our earlier study for the adsorption of per- and polyfluoroalkyl substances (PFAS) [25]. The previously obtained characterization data are briefly summarized here to provide essential background and context for the current adsorption and kinetic investigations.

### 2.2. Experimental Design

Isotherm studies: All the isotherms were performed at pH 6.8 ± 0.2 using 20 g/L of sorbents in 10 mM NaNO_3_ solution as the background electrolyte. The initial contaminant concentrations ranged from 1 to 100 mg/L (1, 10, 25, 50, and 100 mg/L). Control experiments were conducted under identical conditions without the addition of sorbents to account for potential solution-phase losses. Samples were agitated on an end-over-end shaker at 150 rpm and 20 °C for 24 h. After equilibration, suspensions were centrifuged at 3500× *g* for 20 min using an Eppendorf 5804 centrifuge (Hamburg, Germany), and the supernatants were collected for analysis. The residual solids were resuspended in 40 mL of 0.1 mM NaNO_3_ to initiate desorption, which was carried out under the same pH and agitation conditions for 24 h, followed by centrifugation and supernatant collection to quantify the release of As(III) and Co(II).

Kinetic studies: Adsorption kinetics experiments were conducted at an initial metal concentration of 25 mg/L under a pH of 6.8 ± 0.2 and with 10 mM NaNO_3_ as the background electrolyte. Samples were withdrawn at predetermined time intervals (0, 0.2, 0.5, 0.75, 1, 2, 4, and 6 h) to monitor the rate of metal removal. The suspensions were centrifuged at 3500× *g* for 20 min, and the supernatant were collected for analysis. All experiments were conducted in duplicate at 20 ± 1 °C to ensure data reproducibility.

### 2.3. Metal Quantification and Data Analysis

The concentrations of arsenite and cobalt in aqueous samples were quantified using inductively coupled plasma–optical emission spectroscopy (ICP-OES, Agilent Technologies, Wilmington, DE, USA). Instrument calibration was performed with multi-element standard solutions prepared by diluting arsenic oxide in 4% HNO_3_ and cobalt nitrate in 2% HNO_3_ to cover the expected concentration range of the samples. Calibration blanks and quality control standards were analyzed periodically to verify instrument accuracy and precision. Detection wavelengths were selected to minimize potential spectral interferences and ensure analytical reliability. The removal efficacy for both metals was calculated using Equation (1).

Removal (%) = ((C_0_ − C_t_))/C_0_) × 100% (1)

where C_t_ and C_0_ are the concentrations of metals at time t and time zero, respectively.

The equilibrium adsorption behavior of arsenite and cobalt on different sorbents was evaluated using the Langmuir and Freundlich isotherm models. The Langmuir model is expressed as Equation (2) [43]:

(2) $$\frac{1}{q_{e}}=\left(\frac{1}{q_{max}K_{L}}\right)\frac{1}{C_{e}}+\frac{1}{q_{max}}$$

where q_e_ is the denotes the equilibrium adsorption capacity (mg/g), K_L_ (L/mg) is Langmuir’s isotherm constant; q_max_ is the maximum adsorption capacity (mg/g).

The Freundlich equation is formulated as follows Equation (3) [44]:

(3) $$lnq_{e}=\frac{1}{n}C_{e}+lnK_{F}$$

where K_F_ is Freundlich’s constant; and 1/n is the adsorption intensity.

The sorption kinetics of arsenite and cobalt on the three sorbents were evaluated using both pseudo-first-order (PFO) and pseudo-second-order (PSO) models [45]. The governing equations for these kinetic models are provided in Equations (4) and (5).

ln(C_t_/C_o_) = k_1_t (4)

Here, C_0_ represents the initial solute concentration in solution, C_t_ is the concentration at time t, and k_1_ is the rate constant for the pseudo-first-order model.

(5) $$\frac{t}{q_{t}}=\frac{1}{q_{e}}\cdot t+\frac{1}{k_{2}}\cdot \frac{1}{q_{e}^{2}}$$

In this expression, q_e_ denotes the equilibrium sorption capacity, q_t_ is the sorption capacity at a given time t, and k_2_ is the pseudo-second-order rate constant.

The desorption percentage was calculated using Equation (6):

(6) $$Desorption(\%)=\frac{C_{d}V_{d}}{q_{e}m}\times 100$$

where C_d_ is the concentration of the contaminant in the desorption solution (m_g_/L), V_d_ is the desorption volume (L), q_e_ is the adsorption capacity at equilibrium (mg/g), and m is the sorbent mass (g).

Model parameters were obtained from the slope and intercept of the respective linear plots. Linear regression was performed using Excel, and correlation coefficients (R^2^) were used to evaluate the goodness of fit. Model performance was compared based on R^2^ values and visual agreement between experimental and predicted data.

## 3. Results

### 3.1. Characterization of Sorbents

Characterization of the materials is essential for understanding the physicochemical properties of commercially available sorbents, including morphology, porosity, surface area, and functionality. In our previous publication, we performed all the physicochemical characterization of these materials and utilized them for per- and polyfluoroalkyl substances (PFAS) adsorption [25]. The previously obtained characterization data are briefly summarized here to provide essential background and context for the current adsorption and kinetic investigations. Powdered XRD spectra of as-used commercial materials, i.e., FS-100, FS-200, and F-400, were analyzed to understand their crystallographic structure and phase composition. The crystallinity of the materials was determined using powdered XRD analysis, as shown in Figure S1. The sharp peaks in FS-200 and FS-100 confirm the presence of crystalline mineral phases, such as montmorillonite and quartz, in the organic clay. The details of these peaks are described in Table S1. In contrast, the broad peaks in F-400 revealed two broad reflections at approximately 24° and 43.5°, consistent with the disordered microstructure typical of amorphous activated carbon. A distinct, sharp peak observed near 26.55° indicates the presence of minor crystalline graphite inclusions within the carbon matrix [25]. Furthermore, the specific surface area of sorbents is determined using nitrogen gas adsorption–desorption isotherms measured at 77 K by the Brunauer–Emmett–Teller (BET) method. Among them, F-400 exhibited the highest surface area (827 m^2^/g), while the organoclays FS-200 and FS-100 showed comparatively lower values of 106 m^2^/g and 92 m^2^/g, respectively (Figure S2). Barrett–Joyner–Halenda (BJH) pore size distribution analysis indicated that all three materials possess a mesoporous structure, with average pore diameters of approximately 5 nm. Interestingly, the higher surface area of FS-400 did not directly translate to enhanced metal removal efficiency, highlighting that adsorption performance depends not only on surface area but also on pore structure and surface chemical functionality [46,47]. The observed hysteresis loops and BET results collectively confirm that FS-400 exhibits the most favorable textural characteristics, combining an extensive surface area with a well-developed pore network that facilitates molecular diffusion and adsorptive interactions. The surface morphology of the materials is shown in SEM images (Figure S3). The SEM images demonstrated that all the materials exhibited irregular micron-scale particles with heterogeneous morphology. The FS-100 and FS-200 showed a porous structure, but with fewer visible pores. On the other hand, F-400 (Figure S3a,b) exhibited a porous structure and has a high surface area as compared to other sorbents. EDX analysis revealed that FS-100 and FS-200 are predominantly composed of carbon (C), oxygen (O), and silicon (Si), as detailed in Table S2. In contrast, F-400 exhibited a composition primarily dominated by carbon, with minimal contributions from other elemental species.

### 3.2. Sorption Kinetics

**Figure 1:**
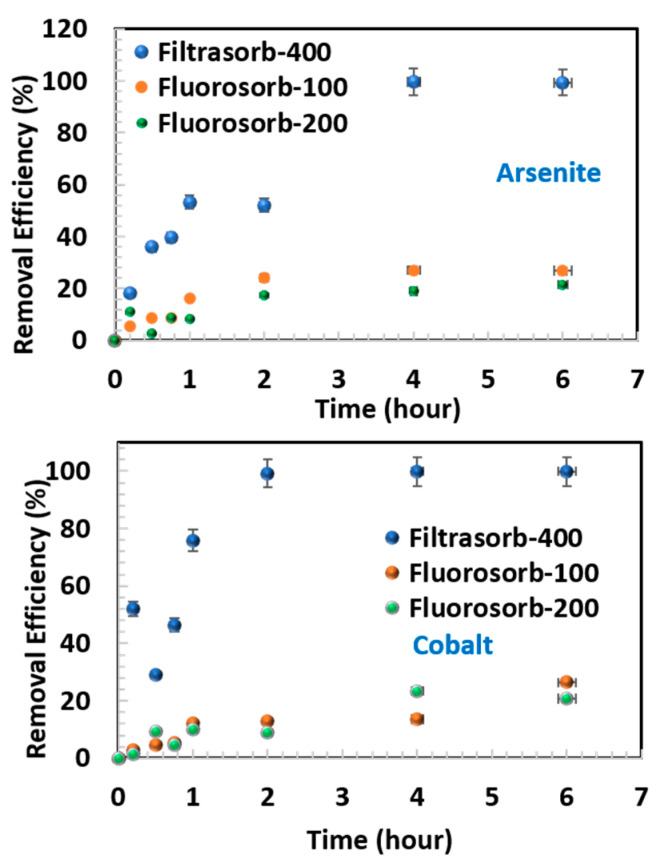
Kinetics of arsenite and cobalt adsorption on all three sorbents.

The sorption kinetics of three sorbents for As(III) and Co(II) were monitored for a period of 6 h (Figure 1). For arsenite, F-400 exhibited rapid adsorption, showing a sharp increasing trend in removal within the first hour and reaching near-equilibrium thereafter. In comparison, FS-100 and FS-200 demonstrated slower removal, characterized by gradual concentration decreases and lower equilibrium capacities. A similar trend was observed for cobalt, where F-400 achieved near-complete removal within 2 h, while FS-100 and FS-200 displayed significantly slower sorption, with considerable residual cobalt remaining in solution after 6 h. These findings highlight the superior kinetic performance of F-400 relative to the organoclay sorbents, suggesting the involvement of distinct sorption mechanisms and surface affinities among the tested materials.

The adsorption kinetics of arsenite and cobalt onto the selected sorbents were analyzed using both pseudo-first-order (PFO) and pseudo-second-order (PSO) models (Figures S4–S6). Given the heterogeneous and porous nature of the sorbents (FS-100, FS-200, and F-400), applying both PFO and PSO models enabled the distinction between whether the adsorption of As(III) and Co(II) is diffusion-limited or mass transfer at active sites. The fitted parameters for the FS-100, FS-200, and F-400 systems are summarized in Table 2, illustrating the distinct kinetic behaviors across the materials. For the F-400, both arsenite and cobalt exhibited stronger correlations with the PSO model (Figure S4), with calculated equilibrium adsorption capacities (qₑ) closely matching the experimental values. This suggests that adsorption, mass transfer at active sites, plays a dominant role in metal uptake on carbon. In contrast, the kinetic fits for FS-100 and FS-200 were less consistent (Figures S5 and S6). Arsenite adsorption on both organoclays aligned more closely with the PSO model, suggesting stronger surface interactions. Conversely, cobalt adsorption on FS-100 and FS-200 followed the PFO model more closely, as indicated by higher correlation coefficients (R^2^), implying that physical adsorption or diffusion-controlled mechanisms predominated. The relatively lower rate constants (K_1_ and K_2_) for the organoclays compared to F-400 further reflect their slower adsorption kinetics. Overall, these results demonstrate that while the PSO model adequately describes arsenite adsorption across all sorbents, cobalt uptake is more complex and cannot be generalized under a single kinetic regime. Cobalt exhibited a gradual increase in sorption capacity across all sorbents, emphasizing the importance of extended contact times for optimal removal, particularly with Fluorosorb-based media.

### 3.3. Effect of Contaminant Concentration on Removal Efficiency

**Figure 2:**
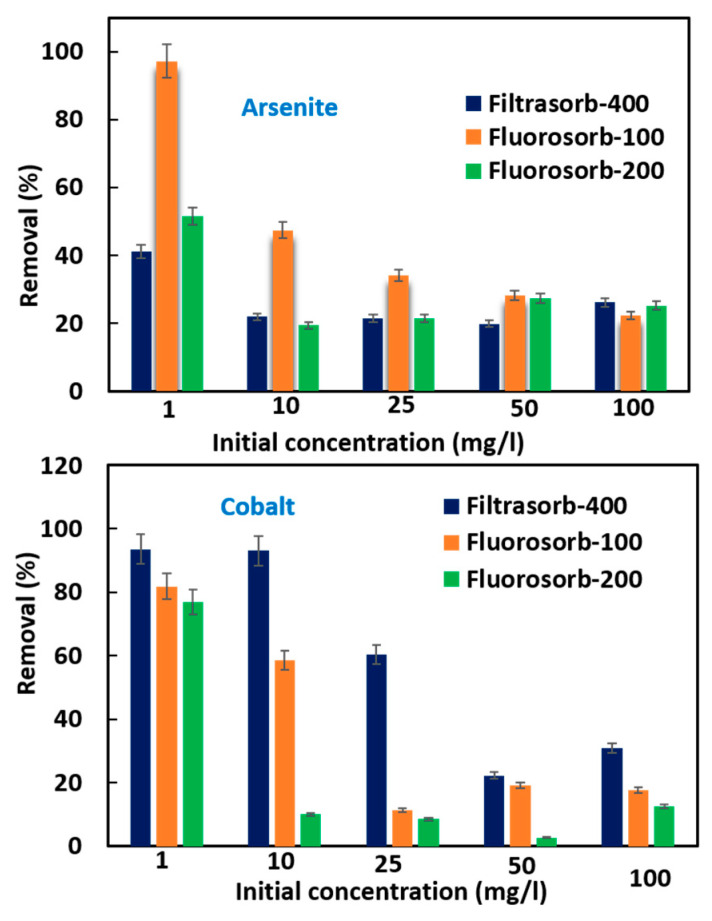
Removal of arsenite and cobalt at various concentration levels.

Removal of cobalt and arsenite was evaluated using FS-100, FS-200, and F-400 across initial concentrations of 1–100 mg/L. At the lowest concentration tested (1 mg/L), cobalt removal exceeded 90% with F-400, while arsenite removal by FS-100 reached over 97%, confirming their strong performance under low-contaminant conditions (Figure 2). As concentrations increased, removal efficiency declined across all sorbents, but the extent of this decrease varied by both contaminant and material. For cobalt, F-400 consistently maintained the highest performance, achieving > 60% removal at moderate concentrations, while FS-100 dropped sharply after 10 mg/L, and FS-200 showed the weakest retention across the range. In contrast, arsenite removal followed a different trend: FS-100 remained the most effective sorbent, sustaining > 25% removal even at 100 mg/L, whereas FS-200 showed moderate efficiencies (~25%) and F-400 declined below this level. These contrasting patterns indicate that F-400 exhibits greater affinity for cationic cobalt, while the engineered surface chemistry of FS-100 enhances the binding of arsenite. Our previous study showed that F-400 exhibited strong carboxylic group peaks (1650–1750 cm^−1^, 1600–1650 cm^−1^, and 1300–1400 cm^−1^), suggesting that oxygenated surface groups while organoclays (FS-200 & FS-100) (broad –OH at 3200–3600 cm^−1^ and alkyl ammonium bands at 2800–3000 cm^−1^) indicate abundant hydroxyls and quaternary-ammonium–modified surfaces [25]. Near-neutral pH (6.8 ± 0.2) in 0.1 mM NaNO_3_, where Co(II) remains as a divalent cation, whereas As(III) is predominantly neutral as H_3_AsO_3_. FS-400, being a granular activated carbon, possesses negatively charged and hydrophobic active surface sites that favor the interaction with cationic species such as Co(II). In contrast, FS-100, an engineered organoclay with modified surface functionalities such as quaternary ammonium, offers a positively charged surface, which facilitates the adsorption of arsenite ions through diffusion and mass transfer [43].

A study by Mandal and Suzuki [48] on the global distribution of arsenic in water reported concentrations ranging from <1 to 48,000 µg/L, with widespread exceedances of the WHO guideline value. At an initial arsenite concentration of ~1 mg/L, removal efficiencies ranged from ~41% with F-400 to ~97% with FS-100, confirming the strong suitability of all three sorbents for environmentally relevant levels. Even under elevated concentrations (25–100 mg/L), each sorbent retained measurable removal efficiency, highlighting their potential applicability for both moderately and highly contaminated waters.

Cobalt concentrations in natural waters are generally very low (<1 µg/L in pristine regions, 1–10 µg/L in inhabited areas, and <1–2 µg/L in drinking water), but can rise markedly in mining and agricultural zones, while seawater typically remains below 1 µg/L [49]. As we observed consistently high cobalt removal across all sorbents, even at ppm-level concentrations, the results highlight their strong effectiveness at environmentally relevant levels. Although removal efficiencies decreased with increasing concentration, the tested sorbents remain highly applicable for cobalt remediation, particularly given their robust performance under comparatively high initial loadings.

### 3.4. Adsorption Isotherms

**Figure 3:**
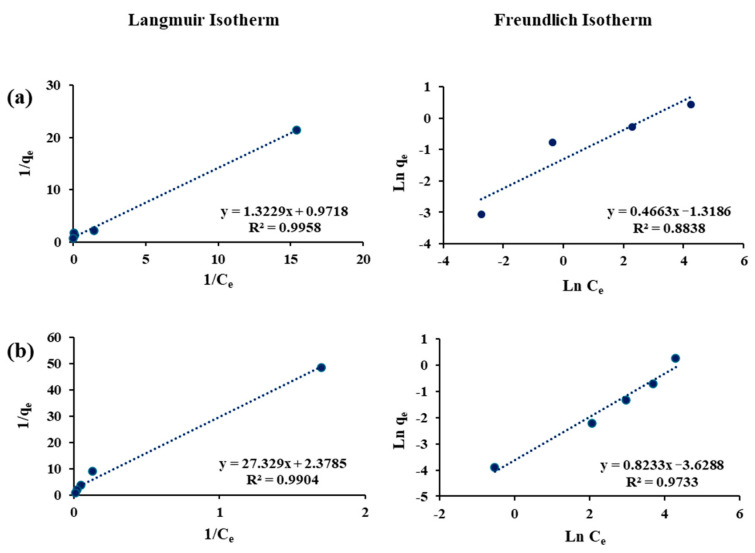
Langmuir and Freundlich isotherm models for the adsorption of (**a**) Cobalt and (**b**) Arsenite on Filtrasorb-400.

**Figure 4:**
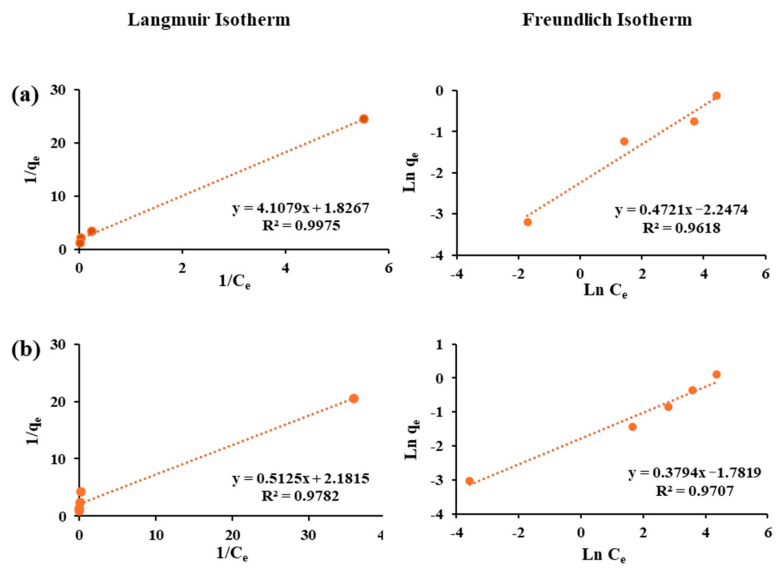
Langmuir and Freundlich isotherm models for the adsorption of (**a**) Cobalt and (**b**) Arsenite on Flurorsorb-100.

**Figure 5:**
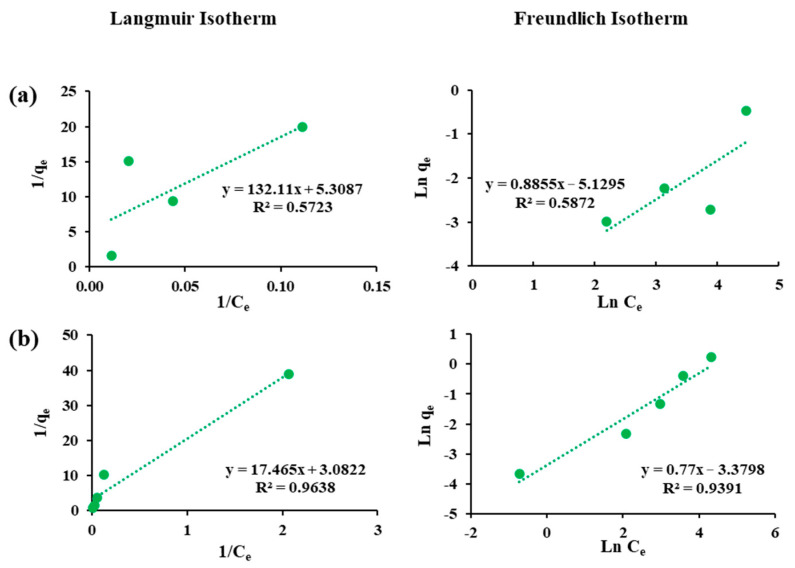
Langmuir and Freundlich isotherm models for the adsorption of (**a**) Cobalt and (**b**) Arsenite on Flurorsorb-200.

The adsorption equilibrium of each sorbent–sorbate pair was evaluated using the linear Langmuir and the Freundlich isotherm models (Figure 3, Figure 4 and Figure 5), and the fitted parameters are summarized in Table 3. For arsenite, both models showed strong correlations, with the Langmuir model generally providing slightly better fits. F-400 exhibited a relatively low maximum adsorption capacity and weak affinity, while FS-200 displayed the lowest overall performance with poor Freundlich correlation (R^2^ = 0.58). In contrast, FS-100 achieved superior uptake (qₘ = 0.46 mg/g) with a high Langmuir constant and favorable Freundlich parameters, suggesting multilayer adsorption on a heterogeneous surface. The superior performance of FS-100 compared to FS-200 can be attributed to its smaller particle size, which provides greater external surface area and shorter diffusion paths, thereby enhancing site accessibility for arsenite adsorption. These findings highlight FS-100 as the most effective sorbent for arsenite removal.

For cobalt, the sorption pattern diverged from that of arsenite. The F-400 showed the highest Langmuir capacity and favorable Freundlich constants, reflecting a strong affinity and efficient removal. FS-100 also performed well for Co(II), supported by consistent Freundlich parameters, while FS-200 showed the weakest uptake. This weaker performance of FS-200, despite its similar chemistry, further underscores the importance of particle size and diffusion limitations in governing adsorption efficiency. The reliability of the adsorption analysis is confirmed by the high correlation coefficients (R^2^ ≥ 0.88) across most fits. Overall, these findings emphasize the contrasting selectivity of the sorbents; FS-100 excels for arsenite removal, while F-400 demonstrates superior affinity toward cobalt.

The relatively low removal efficiency for arsenite of F-400 and FS-200 is consistent with the limited affinity of unmodified carbons and clays; however, performance can be improved by surface oxidation or metal doping. A study on natural well water from Chihuahua (Mexico) reported that raw activated carbon exhibited poor arsenic removal, whereas oxidation treatments improved performance [50]. Moreover, Shi et al. [51] reported that Langmuir capacities of only 2.06–2.98 mg/g for untreated and HNO_3_-modified activated carbon fibers for arsenite, while iron impregnation improved the capacity to 8.65 mg/g, highlighting the role of surface chemistry in enhancing adsorption efficiency. Natural clays are widely recognized as cost-effective sorbents (0.04–0.12 US$/kg compared to ≈21 US$/kg for activated carbon [52] and are utilized in the removal of arsenic from water. The purified natural clay (illite + kaolinite) with a surface area of 128 m^2^/g achieved significant As(III) removal, with a maximum adsorption capacity of 233.1 mg/g at pH 9–10.8 [53]. Adsorption followed the Freundlich isotherm and intraparticle diffusion model, confirming the strong suitability of this clay for arsenite uptake.

On the other hand, cobalt adsorption is strongly influenced by both the surface chemistry of the sorbent and the surrounding solution conditions, as consistently demonstrated in prior studies. For instance, a study demonstrated that cobalt adsorption on activated carbons derived from walnut shells and apple wood was strongly influenced by solution pH, surface chemistry, and the presence of oxygenated functional groups created through oxidation treatments [54]. Moreover, another research using clay minerals reported that kaolinite, montmorillonite, and their acid-activated forms effectively removed Co(II) from water, with adsorption occurring rapidly within 240 min and increasing steadily from pH 1 to 8 [55]. The process was best described by second-order kinetics, with Langmuir capacities ranging from 11.2 to 29.7 mg/g.

### 3.5. Desorption Performance

**Figure 6:**
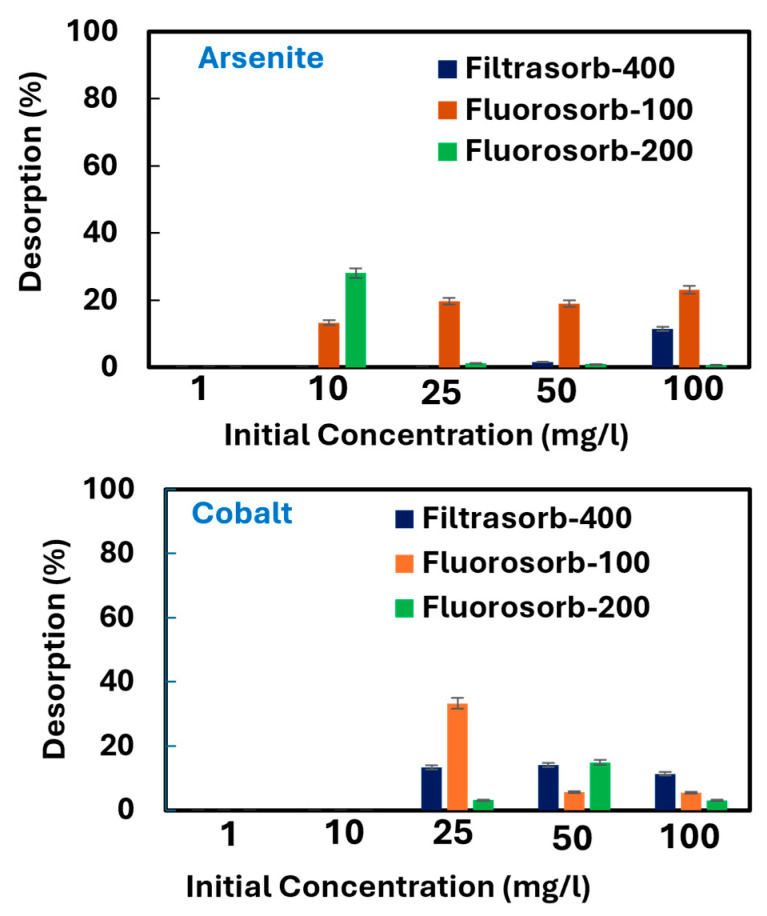
Desorption rates of arsenite and cobalt from all three sorbents.

Desorption studies for arsenite and cobalt were performed under near-neutral conditions (pH 6.8 ± 0.2) across an initial concentration range of 1–100 mg/L. The percentage of desorption for both metals was calculated using Equation (6) (Figure 6), and the mass-normalized release for each concentration level was shown in Tables S3 and S4. The results showed that As(III) was weakly released from all sorbents, indicating largely stable and partially irreversible adsorption. Among the tested materials, FS-100 exhibited the greatest degree of As(III) desorption with increasing concentration, whereas F-400 and particularly FS-200 released comparatively smaller fractions. This trend suggests that the finer particle size of FS-100 facilitates more reversible surface interactions, while the coarser FS-200 and the less modified F-400 provide stronger and more stable retention sites. Similar trends have been observed in other studies, which showed that arsenic desorption from sorbents was minimal, indicating largely stable and partially irreversible adsorption. A study examining the ability of iron acetate-coated activated alumina to remove arsenic As(III) reported that As(III) was largely retained under normal conditions, with alkaline eluents (NaOH) being more effective than acidic eluents (HCl), achieving a maximum desorption of 34.4% using 0.5 M NaOH [56]. Similarly, another study on arsenic desorption from four biochars across pH 4.5–9.5 demonstrated that arsenic was substantially more stable on wood-based biochars, with release rates of only 0.03–2.0%, compared to rice-husk biochars, which released up to 10.4–11.6% at pH 9.5 [57].

In contrast, Co(II) desorption displayed a distinct pattern. F-400 released a moderate but consistent fraction at higher loadings, indicating the coexistence of both reversible and stable binding sites. FS-100 showed greater cobalt desorption at intermediate concentrations, whereas FS-200 exhibited measurable release only at elevated loadings. These observations highlight the differing desorption behaviors of arsenite and cobalt across the sorbents, with F-400 demonstrating more effective cobalt stabilization and FS-200 showing enhanced arsenite retention. Our results are consistent with prior studies that also showed cobalt desorption depends strongly on surface chemistry and the eluent environment. A research study evaluated cobalt recovery from mesoporous activated carbon using hydrochloric, nitric, and sulfuric acids at pH 2.5 [58], supporting the strong binding mechanism.

## 4. Conclusions

The identification of efficient reactive media for arsenite and cobalt removal is essential for developing sustainable water remediation strategies. In this study, three commercially available sorbents, Filtrasorb-400 (F-400), Fluorosorb-100 (FS-100), and Fluorosorb-200 (FS-200), were systematically evaluated for their adsorption and desorption performance under environmentally relevant conditions. All sorbents exhibited high removal efficiencies at near-neutral pH (6.8 ± 0.2) and contaminant concentrations ≤ 1 mg/L. FS-100 demonstrated the highest affinity for arsenite, whereas F-400 showed superior cobalt retention capacity. Desorption experiments confirmed largely irreversible binding, particularly for cobalt on FS-200 and arsenite on F-400, indicating stable sorption interactions. The rapid sorption kinetics, excellent removal efficiencies, and low desorption observed across all three sorbents highlight their strong potential for water treatment applications requiring fast and effective contaminant removal. While this study considered fixed environmental parameters such as pH, ionic strength, and initial contaminant concentration, it did not account for competing anions or hydrophobic organic compounds that may influence metal adsorption. Future work should assess sorbent performance and reusability over multiple cycles under realistic wastewater and groundwater conditions to better evaluate their practical applicability.

## Figures and Tables

**Table 1:** Comparison with other sorbents for the removal of As (III) and Co (II) from aqueous systems.

Adsorbents	Pollutants	pH	Removal (%)	q_max_ (mg/g)	References
Cerium oxide modified AC	As(III)	5	90	36.7	[37]
Modified peanut shell biochar	As(III)	6	86	1.92	[38]
Magnetite nanoparticles	As(III)	8	95	1.63	[39]
Apricot stone activated carbon	Co(II)	9	-	111	[40]
Al-pillared bentonite clay	Co(II)	6		38.6	[41]
Modified Ficus carica leaves	Co(II)	6	57	33.9	[42]

**Table 2:** Kinetic parameters for As(III) and Co(II) adsorption on sorbents.

Adsorbents	Adsorbates	Pseudo-First-Order		Pseudo-Second-Order		
		R^2^	K_1_ (h^−1^)	R^2^	q_e_	K_2_
Filtrasorb-400	As(III)	0.86	0.97	0.90	1.43	0.65
	Co(II)	0.88	1.36	0.96	1.38	1.31
Fluorosorb-100	As(III)	0.76	0.05	0.93	0.39	2.72
	Co(II)	0.88	0.04	0.70	0.35	1.48
Fluorosorb-200	As(III)	0.76	0.03	0.71	0.33	1.92
	Co(II)	0.81	0.04	0.65	0.36	1.24

**Table 3:** Langmuir and the Freundlich isotherm parameters for arsenite and cobalt on sorbents.

Sorbents	Adsorbates	Langmuir Constants			Freundlich Constants		
		R^2^	$q_{m}(mg/g)$	$K_{L}$	R^2^	n	$K_{F}$
Filtrasorb-400	As(III)	0.99	0.42	0.09	0.97	1.20	0.03
	Co(II)	0.99	1.00	0.73	0.88	2.10	0.27
Fluorosorb-100	As(III)	0.97	0.46	4.30	0.97	2.60	0.17
	Co(II)	0.99	0.55	0.44	0.96	2.10	0.11
Fluorosorb-200	As(III)	0.97	0.32	0.04	0.58	1.13	0.01
	Co(II)	0.57	0.19	0.17	0.93	1.30	0.03

## Data Availability

The original contributions presented in this study are included in the article/Supplementary Materials. Further inquiries can be directed to the corresponding author.

## Supplementary Materials

The following supporting information can be downloaded at: https://www.mdpi.com/article/10.3390/ma18225133/s1, Figure S1. XRD spectra of all the sorbents used for the removal of cobalt and arsenic. Figure S2. Nitrogen adsorption- desorption curve for all three commercial sorbents. Figure S3. Representative photograph of the powdered form of commercial sorbents (left) and SEM micrograph of (a,b) F-400, (c,d) FS-100, and (e,f) FS-200 at different magnifications. Figure S4: Pseudo 1st and 2nd order kinetic plots for the adsorption of (a) cobalt and (b) arsenite on Filtrasorb-400; Figure S5: Pseudo 1st and 2nd order kinetic plots for the adsorption of (a) cobalt and (b) arsenite on Flurorsorb-100; Figure S6: Pseudo 1st and 2nd order kinetic plots for the adsorption of (a) cobalt and (b) arsenite on Flurorsorb-200; Table S1: Powdered XRD spectral peaks assignment with corresponding theta values; Table S2: EDS elemental composition of Filtrasorb-400, Fluorosorb-100, and Fluorosorb-200. Table S3. Mass-Normalized Desorption of As(III) from Sorbents; Table S4. Mass-Normalized Desorption of Co(II) from Sorbents.

## Abbreviations

The following abbreviations are used in this manuscript:

FS-200	Fluorosorb-200
FS-100	Fluorosorb-100
F-400	Filtrasorb-400
PRB	Permeable reactive barrier
BET	Brunauer–Emmett–Teller
FE-SEM	Field-emission Scanning Electron Microscopy
XRD	X-ray Diffraction
BJH	Barrett–Joyner–Halenda
PFO	Pseudo-first-order
PSO	Pseudo-second-order
As(III)	Arsenite
Co(II)	Cobalt
EDX	Energy-dispersive X-ray spectroscopy
GAC	Granular activated carbon
ICP-OES	inductively coupled plasma–optical emission spectroscopy
