# Supplementary material for: Adsorptive Removal of Arsenite and Cobalt by Commercial Sorbents
Source: Materials (Basel). 2025 Nov 12;18(22):5133. doi: 10.3390/ma18225133 (PMC12654324; doi:10.3390/ma18225133)
Supplement: Supplementary file 1 [file materials-18-05133-s001.zip › materials-3960330-supplementary.pdf]

Article

Adsorptive Removal of Arsenite and Cobalt by Commercial Sorbents

Sevda Joudiazar [1], Sushma Yadav [1], Zhiming Zhang [2], Anshuman Satpathy [1], Eustace Fernando [1], Roxana Rahmati [1], Junchul Kim [3], Rupali Datta [4] and Dibyendu Sarkar [1,*]

[1] Department of Civil, Environmental and Ocean Engineering, Stevens Institute of Technology, Hoboken, NJ 07030, USA; sjoudiaz@stevens.edu (S.J.); fsushma@stevens.edu (S.Y.); satpathyanshuman92@gmail.com (A.S.); eustace6192@gmail.com (E.F.); rrahmati@stevens.edu (R.R.)
[2] Department of Civil and Environmental Engineering, Rowan University, Glassboro, NJ 08028, USA; zhangz@rowan.edu
[3] Tetra Tech, Inc., King of Prussia, PA 19406, USA; jc.kim@tetratech.com
[4] Department of Biological Sciences, Michigan Technological University, Houghton, MI 49931, USA; rupdatta@mtu.edu
* Correspondence: dsarkar@stevens.edu

Table S1: Powdered XRD spectral peaks assignment with corresponding theta values.

**2θ (Degree)**	**Fluorosorb-100**	**Fluorosorb-200**
7.19◦	Basal spacing of the clay layers	Basal spacing of the clay layers
19.6◦, 26.4◦	Montmorillonite	Montmorillonite
20.2◦, 36.2◦, and 66.06◦	Quartz	Quartz
29.4°	Calcite	Calcite

Table S2. EDS elemental composition of Filtrasorb-400, Fluorosorb-100, and Fluorosorb-200.

Filtrasorb-400		Fluorosorb-100	Fluorosorb-200
Element	Weight (%)	Weight (%)	Weight (%)
C	92.96±0.07	32.33±0.24	25.78±0.3
O	4.69±0.06	39.56±0.27	36.99±0.34
Na	-	0.3±0.05	1.12±0.08
Mg	-	0.88±0.06	1.01±0.08
Al	0.69±0.01	5.77±0.1	7.67±0.16
Si	0.93±0.02	15.02±0.17	20.86±0.27
S	0.72±0.02	-	-
N	-	1.07±0.18	0.83±0.22
Fe	-	5.07±0.37	5.73±0.48

Table S3. Mass-Normalized Desorption of As(III) from Sorbents.

Initial Concentration (mg/L)	Mass-Normalized Release of As (III) (mg/g)		
	F-400	FS-100	F-200
10	0.39	0.23	0.45
25	0.98	0.74	0.64
50	2.00	1.66	1.13
100	3.55	3.64	2.70

Table S4. Mass-Normalized Desorption of Co(II) from Sorbents

Initial Concentration (mg/L)	Mass-Normalized Release of Co (II) (mg/g)		
	F-400	FS-100	F-200
10	0.03	0.21	0.43
25	0.39	1.06	0.82
50	1.86	2.00	1.47
100	3.28	4.07	3.36

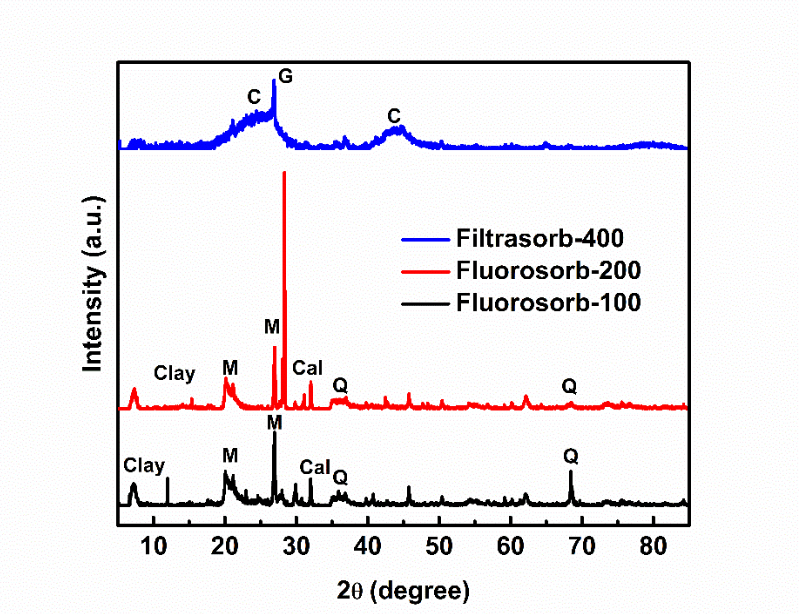

Figure S1. XRD spectra of all the sorbents used for the removal of cobalt and arsenic.

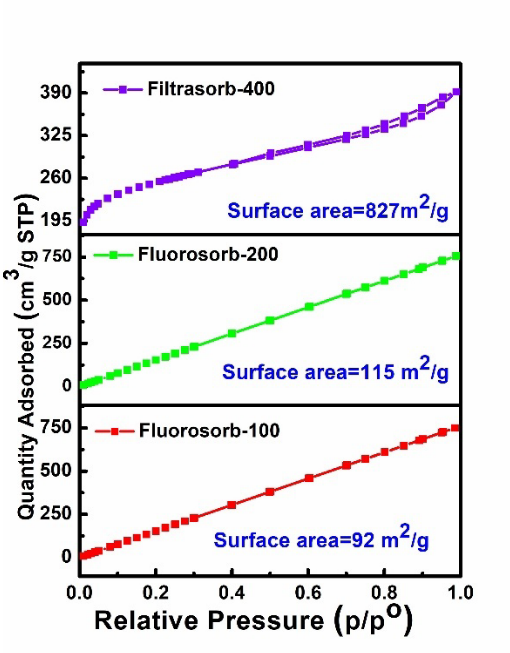

Figure S2. Nitrogen adsorption- desorption curve for all three commercial sorbents.

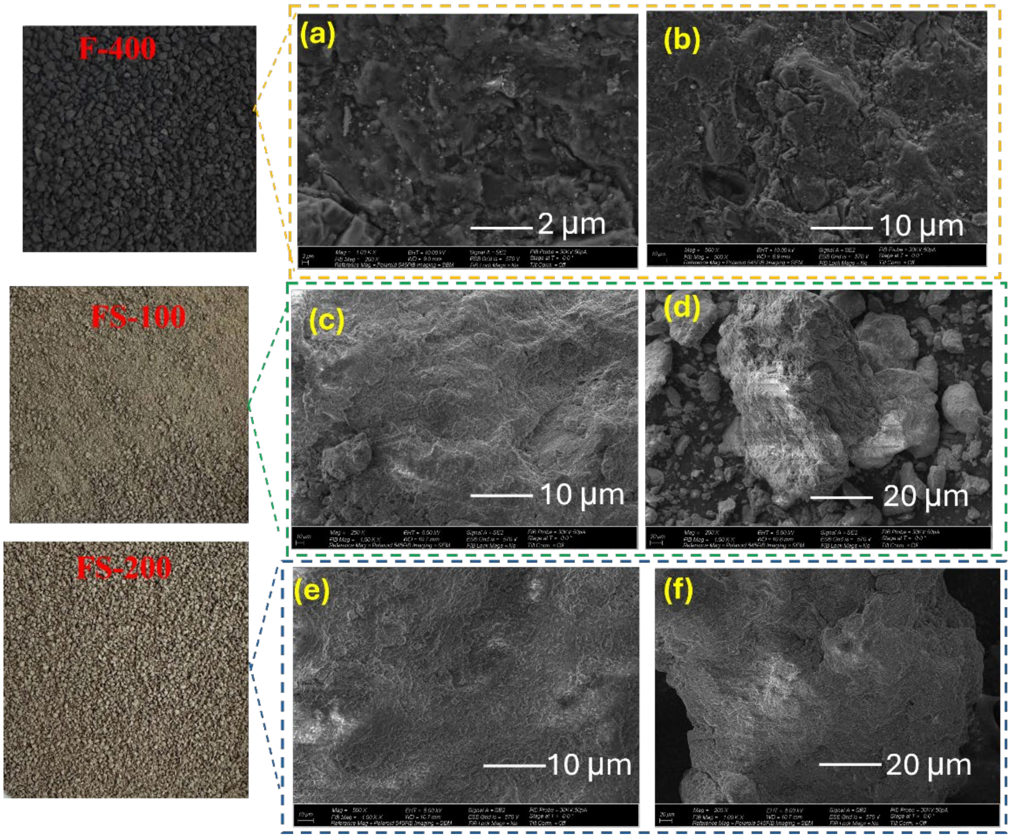

Figure S3. Representative photograph of the powdered form of commercial sorbents (left) and SEM micrograph of (a,b) F-400, (c,d) FS-100, and (e, f) FS-200 at different magnifications.

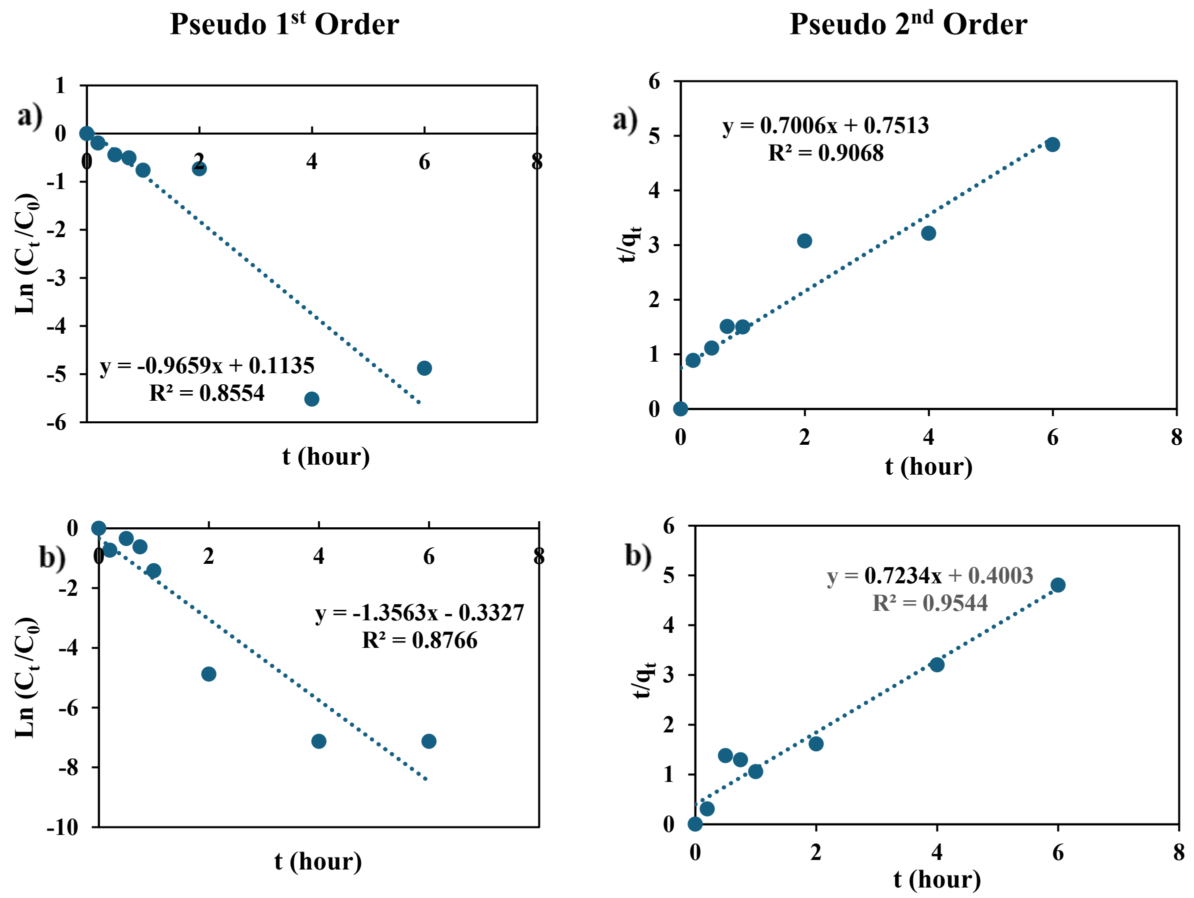

Figure S4. Pseudo 1st and 2nd order kinetic plots for the adsorption of **(a)** cobalt and **(b)** arsenite on Filtrasorb-400

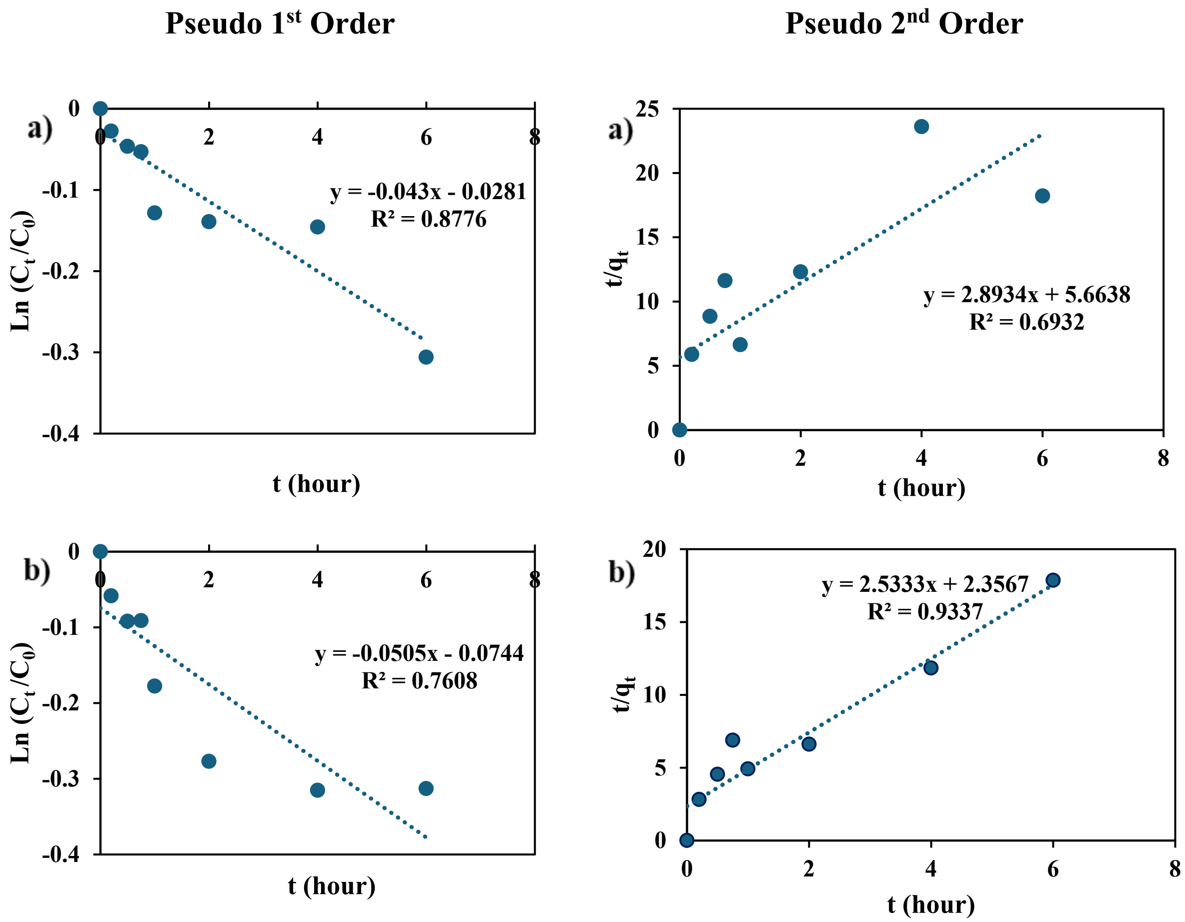

Figure S5. Pseudo 1st and 2nd order kinetic plots for the adsorption of **(a)** cobalt and **(b)** arsenite on Flurorsorb-100

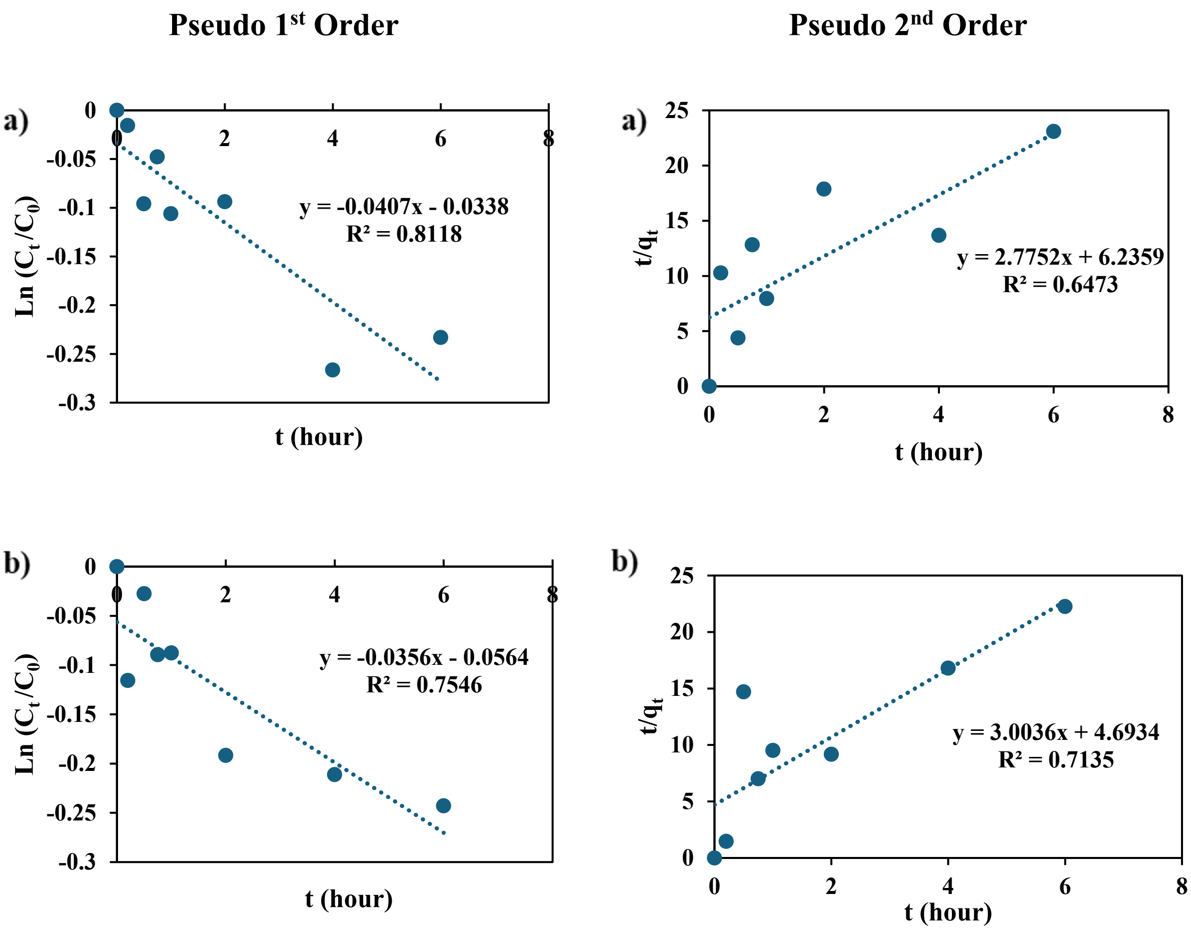

Figure S6. Pseudo 1st and 2nd order kinetic plots for the adsorption of **(a)** cobalt and **(b)** arsenite on Flurorsorb-200
